# Paraventricular Nucleus Sim1 Neuron Ablation Mediated Obesity Is Resistant to High Fat Diet

**DOI:** 10.1371/journal.pone.0081087

**Published:** 2013-11-19

**Authors:** Dong Xi, Jeff Roizen, Meizan Lai, Nilay Gandhi, Bassil Kublaoui

**Affiliations:** Division of Endocrinology and Diabetes, The Children's Hospital of Philadelphia, University of Pennsylvania Perelman School of Medicine, Philadelphia, Pennsylvania, United States of America; University of Santiago de Compostela School of Medicine - CIMUS, Spain

## Abstract

Single minded 1 (SIM1) is a transcription factor involved in brain patterning and control of energy balance. In humans, haploinsufficiency of SIM1 causes early-onset obesity. Mice deficient in the homologous gene, SIM1, also exhibit early onset obesity and increased sensitivity to a high fat diet. SIM1 is expressed in several areas of the brain implicated in control of energy balance including the paraventricular nucleus (PVN), the supraoptic nucleus (SON), the medial amygdala and nucleus of the lateral olfactory tract. We have previously shown that mice with global Sim1 neuron ablation exhibit obesity with hyperphagia as the primary defect. The PVN has a critical role in feeding and in high-fat appetite, thus, we sought to determine the effect of Sim1 neuron ablation limited to the PVN. We achieved PVN-SIM1 limited ablation through stereotactic injection of diphtheria toxin into the PVN of Sim1Cre-iDTR mice. The specificity of this ablation was confirmed by immunohistochemistry and quantitative real time PCR of the PVN, supraoptic nucleus and the amygdala. Mice with PVN Sim1 neuron ablation, similar to mice with global Sim1 neuron ablation, exhibit early onset obesity with hyperphagia as the primary defect. However, PVN-Sim1 neuron ablated mice have a decreased response to fasting-induced hyperphagia. Consistent with this decrement, PVN-Sim1 neuron ablated mice have a decreased hyperphagic response to PVN injection of agouti-related peptide (AgRP). When PVN-Sim1 neuron ablated mice are placed on a high fat diet, surprisingly, their intake decreases and they actually lose weight. When allowed ad lib access to high fat diet and normal chow simultaneously, PVN-Sim1 neuron ablated mice exhibit overall decreased intake. That is, in PVN-Sim1 neuron ablated mice, access to fat suppresses overall appetite.

## Introduction

Energy homeostasis is maintained by multiple mechanisms that integrate information on the body’s nutritional status to induce behavioral and metabolic responses to changes in fuel availability [1,2]. Previous work has shown that electrolytic lesioning of the entire paraventricular nucleus (PVN) induces hyperphagia on a normal diet [3]. Normal PVN development is dependent on SIM1, a brain patterning transcription factor [4]. Mice with complete (homozygous) or partial (heterozygous) deficiency of SIM1 have hyperphagic obesity with increased sensitivity to a high fat diet [5-8]. Global ablation of Sim1 neurons, similar to complete deficiency of SIM1, induces obesity that appears to occur due to hyperphagia as a primary defect [9]. In addition to the PVN, SIM1 is expressed in other areas of the brain implicated in energy homeostasis including the supraoptic nucleus (SON), the medial amygdala and the nucleus of the lateral olfactory tract (NLOT) [10]. 

Signaling through the melanocortin receptor 4 (MC4R) in the PVN is critical in normal energy balance [11-14]. MC4R appears to have a specific role in regulating the homeostatic response to dietary fat, evidenced by the profound fat-induced hyperphagia in MC4R ^-/-^ mice [15]. Mice with a deficiency in either the SIM1 gene or of MC4R show phenotypic similarities, both exhibiting early-onset hyperphagia, increased linear growth and enhanced sensitivity to a high-fat diet [6,16]. Administration of melanocortin agonists in rodents reduces intake of normal chow by decreasing meal size [12,15,17]. Although they appear to act at the same anatomic locus, the relationship between SIM1 related pathways and MC4R signaling in the PVN remains uncharacterized. 

Given the role of the PVN in regulating intake, we sought to examine if PVN-Sim1 neuron ablation was sufficient to recapitulate the effects of global Sim1 neuron ablation. We found that mice with PVN-Sim1 neuron ablation, similar to mice with global Sim1 neuron ablation, exhibit early onset obesity with hyperphagia as the primary defect. However, PVN-Sim1 neuron ablated mice have a decreased response to fasting-induced hyperphagia. Consistent with this decrement, PVN-Sim1 neuron ablated mice have a decreased hyperphagic response to PVN injection of Agouti-related peptide (AgRP). When PVN-Sim1 neuron ablated mice are placed on a high fat diet, their intake decreases and they actually lose weight. When allowed ad lib access to high fat diet and normal chow simultaneously, PVN-Sim1 neuron ablated mice exhibit overall decreased intake. 

## Materials and Methods

### Animals

All procedures were carried out in accordance with the National Institutes of Health Guidelines on the Care and Use of Animals and approved by the Children’s Hospital of Philadelphia Institutional Animal Care and Use Committee (Protocol #2009-10-895).

6 to 8 week old mice were used in our studies. Sim1creiDTR (diphtheria toxin receptor) mice were generated by crossing heterozygous Sim1cre with homozygous iDTR mice, as described previously [9]. Sim1cre mice have been previously characterized and were obtained from Dr. Joel Elmquist [10]. Homozygous iDTR mice were obtained from Jackson laboratories (stock number 007900, The Jackson Laboratory, Bar Harbor, ME). iDTR mice express the simian diphtheria toxin receptor downstream of a loxp flanked stop cassette under control of the ROSA26 locus. Thus expression of iDTR occurs only in the presence of Cre recombinase. Binding of exogenous diphtheria toxin (DT) to the iDTR causes neuronal apoptosis. Experiments were performed on Sim1creiDTR mice and littermate iDTR controls. Animals were genotyped by multiplex PCR as previously described[9]. for each genotype with the following primers: 5’ –cacgaccggcaaacggacagaa-3’, 5’-tgggattagcgtgtttcaactgagc-3’, 5’-ttttggttttggatgagtctgtggag-3’. 

### PVN cannulation and injection of diphtheria toxin (DT)

PVN cannulation and injection of DT was performed as previously described[18]. Briefly, mice were anesthetized with isoflurane (2%, 2L O2/min). Using a stereotaxic apparatus (David Kopf Instruments, Tujunga, CA) a guide cannula (Plastic One Inc., Roanoke, VA) was affixed to the skull to extend 0.22mm caudal to bregma, 0.25mm lateral to midline, and 4.6 mm below the surface of the skull. To ablate Sim1 neurons bilaterally, Mice were injected into each side of the PVN with 1.0ng DT in 200 nl via a 5.5 mm internal cannula. Previous work has shown that this amount is appropriate to specifically ablate Sim1 neurons in the PVN but not in other nuclei. Injection was performed with a syringe pump (Cat. 703006, Harvard apparatus, Holliston, MA) over a period of 4 min. After delivery of DT, the internal cannula was left in place for 4-5 minutes to prevent reflux, and then slowly withdrawn. 

### Feeding, growth, fasting and AgRP and melanocortin-4-receptor agonist injection studies

Prior to surgery all mice were kept on a normal chow diet (PicoLab Mouse Diet 20 from Labdiet, Elkridge, MD) containing 3.75kcal/g (10% fat). They were on a 12:12 light cycle with lights on at 6AM. After mice underwent surgery and intra-PVN DT injection, body weight and food intake were measured weekly as previously described [8]. Energy expenditure was measured using the Comprehensive Laboratory Animal Monitoring System (Columbia Instruments, Columbus, OH) 6 weeks after injection. Metabolic challenge studies were performed 6 weeks after DT injection. Mice were randomly selected from all cohorts, and Sim1creiDTR, iDTR mixed-gender mice were involved in food intake assessment. After the injection of 200 nl aCSF into the PVN at 2 PM, 4 hours of light cycle food intake and 24 hours of overall intake were measured. The following day, mice were deprived of food from 5 PM to 9 AM. At 9 AM, mice were re-fed with normal chow diet and food intake was recorded at 4 hours and 24 hours. These mice were allowed two days to recover. Then, at 9 AM, these mice were injected with 20 pmol AgRP (Phoenix Pharmaceutical Inc.) or 20 pmol MC4R selective agonist (cyclo(α-Ala-His-D-Phe-Arg-Trp-Glu)-NH2)(Bachem, US) in 200 nl aCSF over 4 min as above. Food intake was recorded at 4 hours or 24 hours, or energy expenditure was monitored at (for) 30 min, 1 hour, 2 hours, 3 hours and 4 hours after the injection. 

### Food intake, HF feeding and food choice studies

Six weeks after injection, mice were maintained on normal chow diet, then for one week they were switched for a week to a high-fat diet (DIO (VHFD) 60 kcal% fat, D12492, Research Diet, NJ), and then were switched back to normal chow diet. Food intake was measured daily and overall and per kg energy intake was calculated on the high fat diet, as well as on the normal chow diet. After a week of recovery on normal chow diet, food choice was measured; for this experiment equal amounts of normal chow and high fat pellets were placed on the bottom of the cage and weighed daily. 

### Immunohistochemistry

Brain samples were prepared and immunofluorescence staining was performed as previously described with minor modifications described below [7,19,20]. Briefly, at 16 weeks of age Sim1creiDTR and iDTR mice (mixed gender) were anesthetized with isoflurane and transcardially perfused with heparinized 0.9% saline, followed by 4% paraformaldehyde (PFA). The brain was removed and sectioned coronally into 30 µm sections using a sliding microtome (Leica SM 2000R, Buffalo Grove, IL). Sections containing PVN, SON were blocked with 3% goat serum then incubated overnight at 4°C with rabbit anti- SIM1 antibody (AB4144, Millipore Corp., Billerica, MA) diluted 1:1000 in 3% goat serum. Sections were then washed with 0.3% Triton-X, incubated for 2 hours at room temperature with CY3 goat anti-mouse secondary antibody or CY3 goat anti-rabbit secondary antibody (115-165-166 , or 111-165-003 Jackson ImmunoResearch Laboratories, West Grove, PA) at 1:400. Sections were mounted onto slides with vectashield mounting medium with DAPI (H-1200, Vector Laboratories, Burlingame, CA). Images containing PVN and SON were captured using DAPI and CY3 channels under an Olympus BX61 microscope (Center Valley, PA) with Cytovision software (Applied Imaging Corp., San Jose, CA). Cell counting was performed using ImageJ software. The contrast of images was adjusted and the area where the specific nucleus is located was defined as AOI (area of interest). The particles in the AOI were counted by setting the same threshold for both groups. 

### Quantitative RT-PCR

qPCR was performed as previously described with minor modifications as described below [7]. Briefly, PVN and amygdala were dissected from fresh brains using a mouse brain block (David Kopf instruments, Tujunga, CA). Total RNA was extracted using Tripure reagent (Roche Applied Science, Indianapolis, IN). cDNA was synthesized as previously described[8]. Duplex qPCR was performed using an ABI step-one plus real-time PCR system (Applied Biosystems, Foster City, CA) and Taqman assays for the following genes using beta-actin (Cat. No. 4352341E, Applied Biosystems, Foster City, CA) to normalize for total mRNA used. Each qPCR experiment was repeated using GAPDH as the control, with similar results. The following probes were used: SIM1 (Mm00441390_m1), MC4R (Mm00457483_s1), NPY (Mm03048253_m1), AgRP (Mm00475829_g1), OT (Mm00726655_s1).

### Statistics

All values are presented as Mean ± SEM and represent data from a minimum of two repeated experiments. Feeding efficiency was calculated as the ratio between weekly body weight change and weekly food intake. Energy intake was calculated by multiplying gram food intake by calorie per gram food. Data was analyzed using Prism Software (GraphPad Software, San Diego, CA). Means were compared using a two-tailed t-test, with Welch’s correction or repeated measures two-way ANOVA with Bonferroni post-tests. Differences were considered statistically significant if p < 0.05 (*) and p< 0.01 (**). 

## Results

### Generation and Analysis of mice with specific ablation of PVN Sim1 neurons

**Figure 1:**
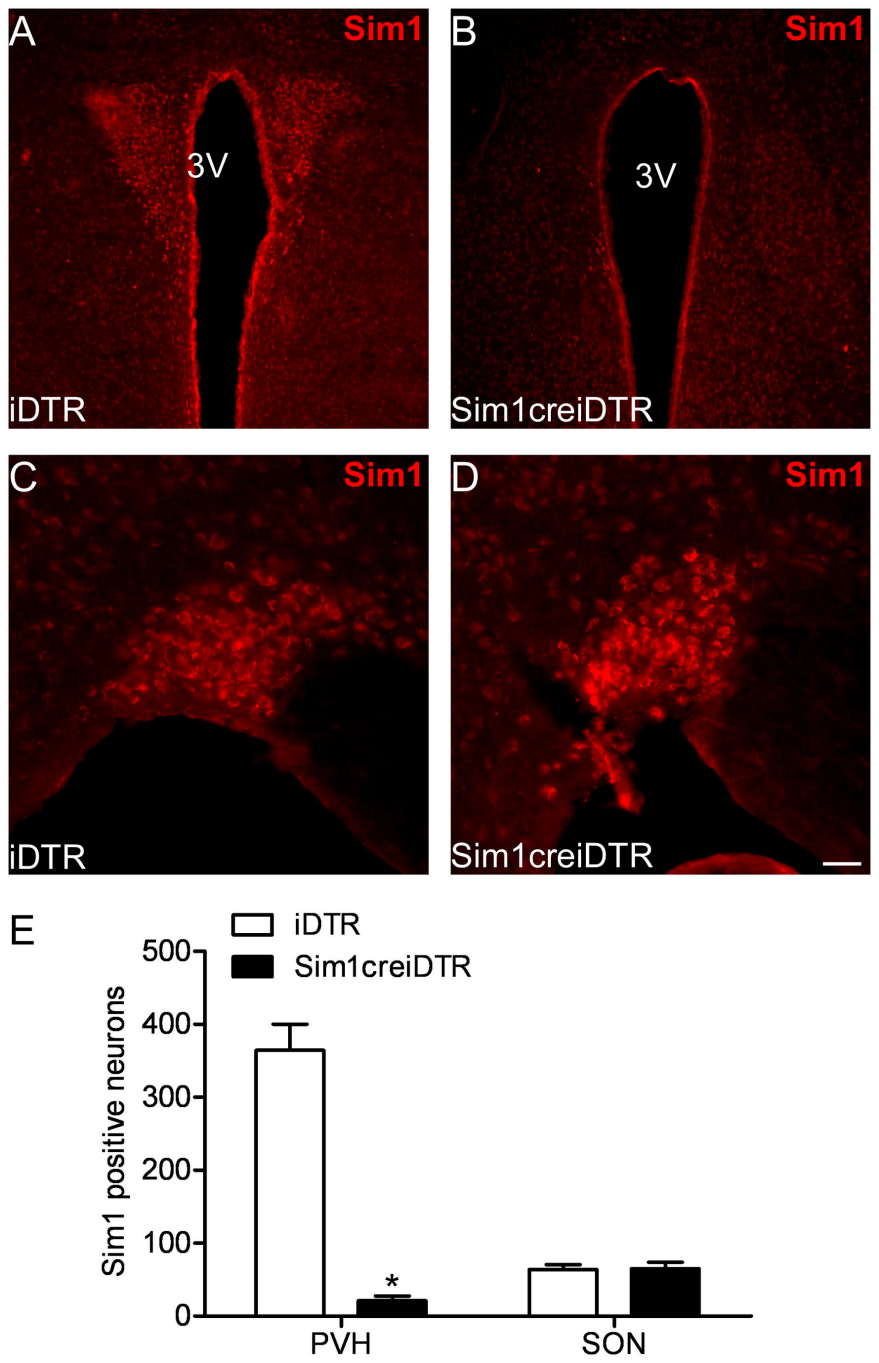
Immunofluorescence and quantitation of *SIM1* neurons in PVN and SON of iDTR and Sim1Cre mice. (A) PVN of iDTR mice shows robust expression of *SIM1*, (B) *SIM1* staining was dramatically decreased in PVN of Sim1creiDTR mice. Robust expression of *SIM1* was observed in SON of both iDTR (C) and Sim1creiDTR mice (D). (E) Quantitation of *SIM1* positive neurons in PVN and SON reveals similar numbers of *SIM1* positive neurons in SON of both iDTR and Sim1Cre mice but a significant decrease in SIM1 neurons of PVN of Sim1Cre mice relative to iDTR mice (n=3 for each group, * p<0.05). Scale bar: 40 µm for A, B; and 20 µm for C, D.

**Figure 2:**
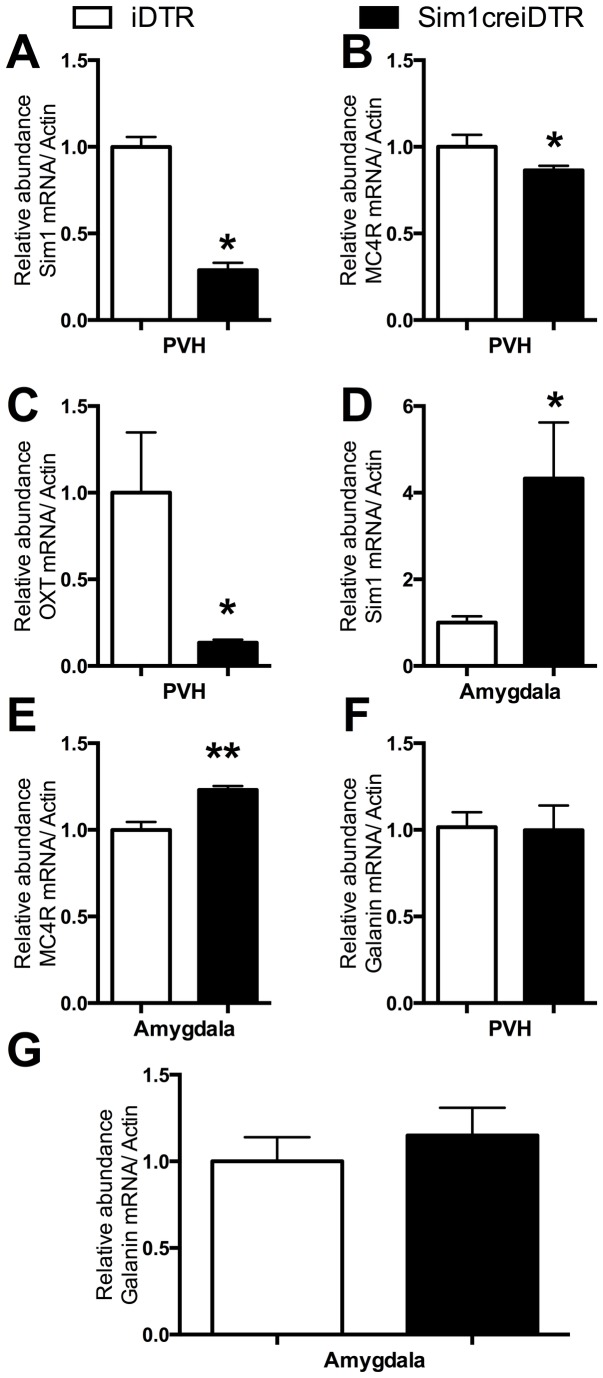
Body weight (A)(D), food intake (B)(E) and feeding efficiency of male and female Sim1creiDTR and iDTR mice. Body weight and food intake were measured weekly on a chow diet (n= 10 for male groups, n=9 for female groups, *p<0.05) (C) (F) feeding efficiency of male and female mice calculated as the ratio between weekly body weight change (g) and food intake (g) (n=8 for each group, * p<0.05).

To specifically ablate Sim1 neurons in the PVN, we performed bilateral PVN cannulation and diphtheria toxin (DT) injection of Sim1creiDTR and iDTR control (Sim1cre negative and iDTR positive) mice. To assess the extent and specificity of Sim1 neuron ablation, immunofluorescence staining for Sim1 was performed in all mice by counting SIM1 positive neurons in the PVN and SON. Sim1creiDTR post-injection mice displayed a significant reduction in number of Sim1 neurons in the PVN (Figure 1A, B), but not in the SON (Figure 1 C,D). Cell counting and analysis showed that number of SIM1 positive neurons was significantly decreased in the PVN (364.6 ± 35.68 for iDTR, and 21.17 ± 6.667 for Sim1creiDTR), but not significantly changed in the SON (63.75 ± 6.448 for iDTR, and 65.0 ± 6.794). To assess SIM1 mRNA content, we performed qPCR on tissue punches from Sim1creiDTR and iDTR control mice after PVN delivery of DT. Overall SIM1 mRNA abundance in Sim1creiDTR mice was decreased by 80% in the PVN relative to iDTR littermates (Figure 2A). 

### Specific PVN Sim1 Neuron Ablation Causes Hyperphagic Obesity

**Figure 3:**
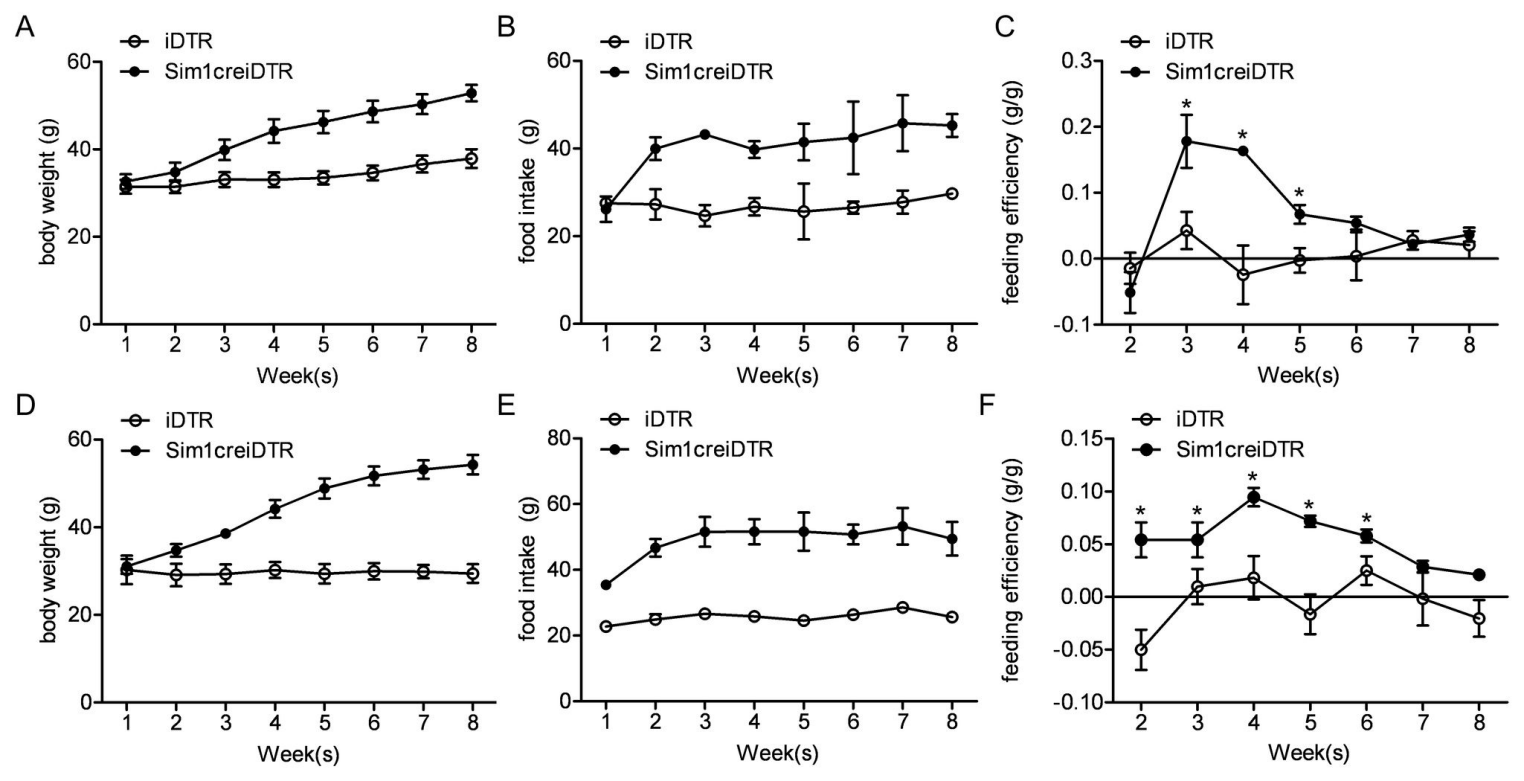
Food intake after 12 hour fast, food intake after AgRP injection and metabolic rate after MC4R agonist injection. (A) (B) 4 hour and 24 hour intake of normal chow after fasting in Sim1creiDTR and iDTR mice after overnight 12 hour food deprivation (n≥4 for each group). (C)(D) 4 hour and 24 hour intake of normal chow in Sim1creiDTR and iDTR mice after injection of AgRP at 20 pmol into the PVN (n≥4 for each group). (E)(F) Oxygen consumption and metabolic rate of Sim1creiDTR and iDTR mice at 0min, 15 min, 30min, 60 min, and 120 min after injection of MC4R selective agonist into the PVN (n=3 for each group). Energy expenditure was measured in CLAMS cages during light cycle. *p< 0.05, **p<0.01, ***p<0.001.

Prior to specific ablation of PVN neurons there was no significant difference in weight between Sim1creiDTR mice and iDTR control mice. Starting two weeks after ablation, body weight of both male and female PVN Sim1 neuron ablated mice diverged from controls (Figure 3 A,D). At sacrifice at 14 weeks of age, the weight of PVN Sim1 neuron ablated mice was statistically significantly increased by 107% in females (P <0.05) and 130% in males (P <0.05). This increase in body weight was significantly correlated with an increase in food intake (Figure 3 B,E) in both male and female PVN Sim1 neuron ablated mice (R^2^=0.8425). Similar to mice with global SIM1 ablation, feeding efficiency (grams of food intake per gram of weight) was significantly higher in PVN Sim1 neuron ablated mice than iDTR controls overall, peaking two weeks post -DT injection (Figure 3 C,F). 

### PVN Sim1 Neuron Ablated mice have a decreased hyperphagic response to food deprivation, however, this decrease may reflect a ceiling effect for food intake

**Figure 4:**
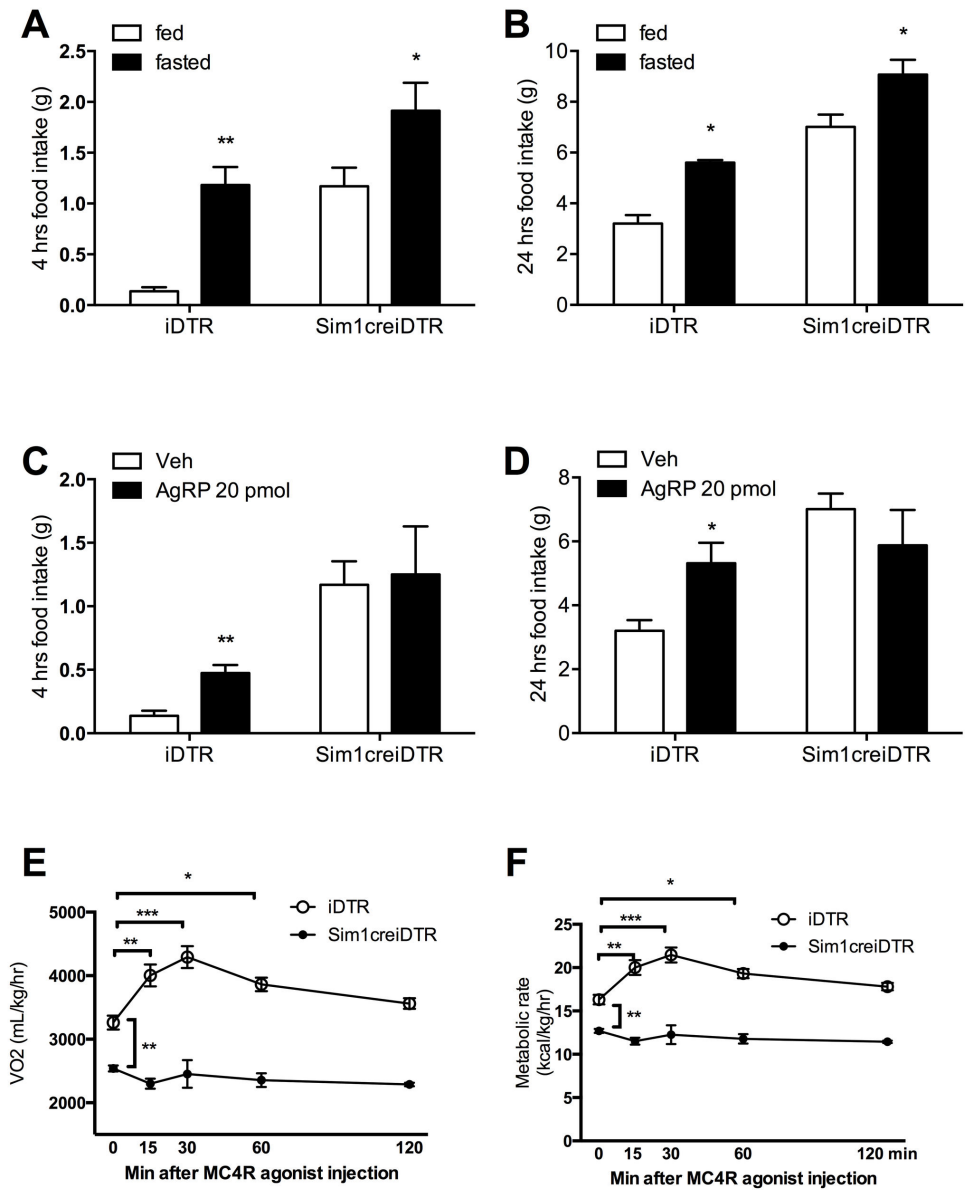
Response of Sim1creiDTR and iDTR mice to high fat diet. (A) Food intake and (B) energy intake of Sim1creiDTR and iDTR mice. Mice were fed with chow for a week, switched to HF for a week, then back to chow(n=6 for each group). (C) Average daily body weight change of Sim1creiDTR and iDTR mice when fed with a chow diet or HF diet (n=6 for each group). (D)(E)Daily food intake (E) and energy intake (F) of Sim1creiDTR and iDTR mice fed with mixed diet of chow and HF (n=7 to 9 for each group). * p<0.05.

To assess the response of mice with PVN Sim1 neuron ablation to fasting, Sim1creiDTR and iDTR mice were subjected to 12 hours of food deprivation and food intake was monitored for four hours during refeeding. Compared to unfasted iDTR controls, fasted iDTR mice increased their food intake by 766% (p< 0.05) during 4 hours of re-feeding (Figure 4A, B). By contrast, fasted PVN Sim1 neuron ablated mice only increased their four-hour food intake by 64% (p< 0.05). The difference in percent increase between these groups was statistically significant (p< 0.05), and remained statistically significant at 24 hours (29% increase in Sim1icreDTR vs 75% increase in iDTR mice; p<0.05). This result is consistent with the possibility that PVN Sim1 neuron ablated mice exhibit a blunted response to food deprivation. It is possible, however, that the Sim1CreiDTR mice may have reached a ceiling in their possible intake post-fasting and so the meaning of the difference in relative increase in food intake is not entirely clear.

### PVN Sim1 Neuron Ablation leads to blunted response to MC4R signaling

Previous work has shown that melanocortin agonists increase food intake and energy expenditure when directly injected into normal rat PVN, presumably via action at MC4R [21-24]. Recent work has revealed some of the circuitry underlying this signaling with AgRP neurons being found to project to Sim1 neurons in the PVN to mediate food intake[25]. Thus, to investigate the possibility that PVN Sim1 neuron ablation decreases MC4R signaling, we examined 1) food intake following injection of AgRP and 2) energy expenditure following injection of MC4R selective agonist into the PVN. 

After injection of 20 pmol AgRP, iDTR mice showed a significant increase in 4-hour food intake (348% Figure 4C, p<0.05), while 4-hour food intake of PVN Sim1 neuron ablated mice was not significantly changed relative to vehicle injected controls. Similarly, 24-hour food intake following the AgRP treatment increased by 66% in iDTR mice (p<0.05), compared with a non-significant decrease in Sim1 PVN neuron ablated mice (Figure 4D). At baseline, VO_2_ (Figure 4E) and metabolic rate (Figure 4F) of iDTR mice were significantly different than PVN SIM1 neuron ablated mice (Figures 4E and 4F – for baseline, p<0.01). Following injection of an MC4R selective agonist into the PVN, VO_2_ (Figure 4E) and metabolic rate (Figure 4F) of iDTR mice were significantly increased, at 15 minutes by 21% (p<0.05), at 30 minutes by 31% (p<0.01), and at 1 hour by 18%, but was not significantly different by 2 hours after injection, while PVN SIM1 neuron ablated mice did not show any significant changes from baseline at these same time points. Thus, PVN Sim1 neuron ablation leads to decreased sensitivity to both the appetite inducing as well as the energy expenditure increasing effects of MC4R signaling. 

### PVN Sim1 Neuron Ablation leads to resistance to High Fat Diet induced-hyperphagia

**Figure 5:**
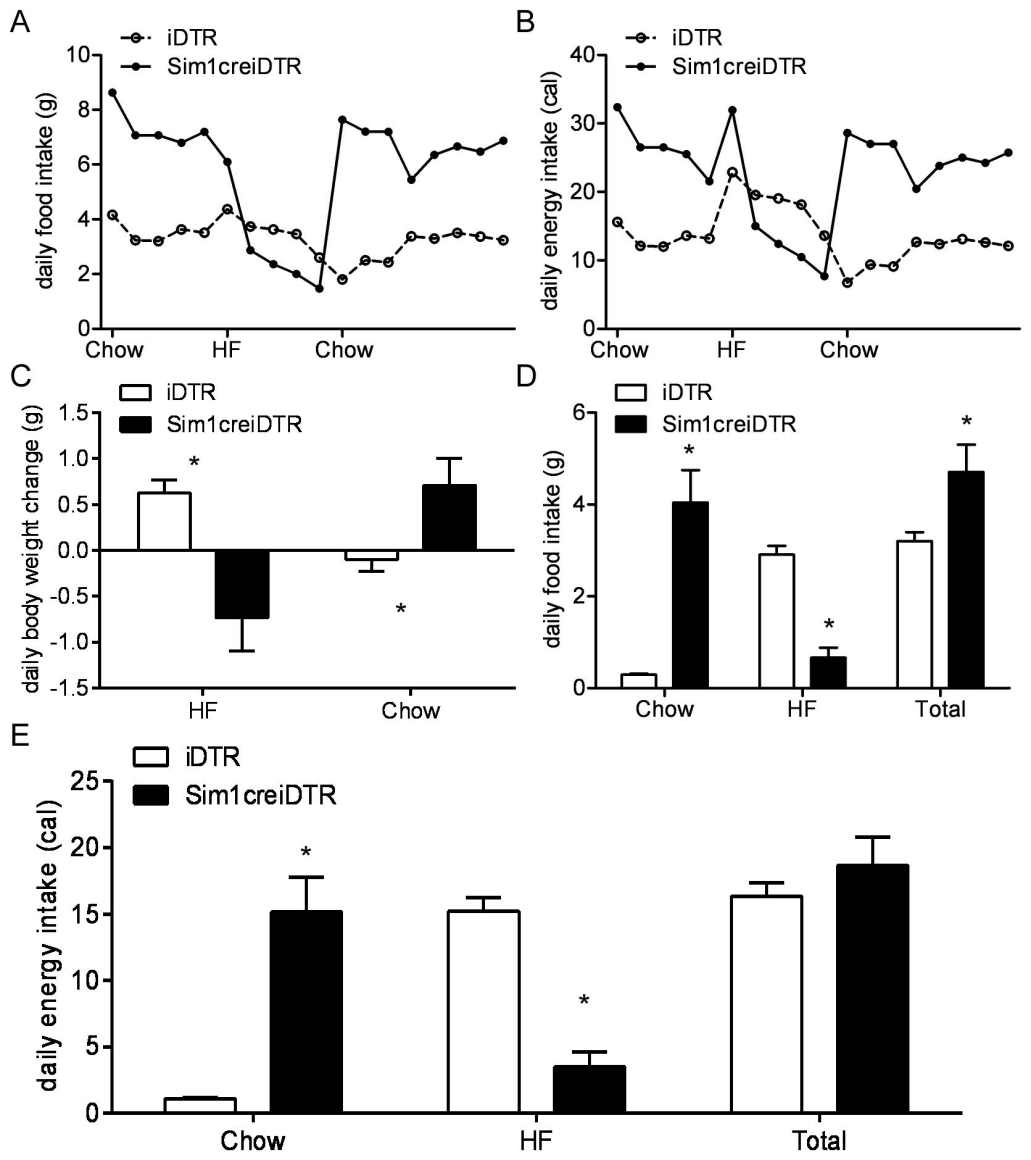
Real-time quantitative PCR comparing Sim1 mRNA, MC4R mRNA, and Galanin mRNA in PVN and Amygdala of Sim1creiDTR and iDTR mice, and comparing OXTmRNA in PVN of Sim1creiDTR and iDTR mice. Relative mRNA abundance of *SIM1* (A), MC4R (B), OXT (C), and GAL (F) expression in the PVN and *SIM1* (D), MC4R (E), and GAL (G) amygdala. (n=4 to 9 for each group, * p< 0.05, ** p<0.01, ***p<0.001) .

Mice with global haploinsufficiency of SIM1 (SIM1^+/-^mice) become obese when fed a HF diet [6]. To investigate the role of PVN Sim1 neurons in HF diet induced obesity, we examined daily food intake of PVN Sim1 neuron ablated mice before and after switching from normal chow diet to a HF diet. In this paradigm, mice were maintained on normal chow diet, then switched to a high fat diet for a week and then returned to normal chow diet (Figure 5A, B). As expected, in iDTR mice, a switch to a HF diet induced an increase in caloric intake that peaked on day one and then declined to a steady state value slightly higher than caloric intake on normal chow. Conversely, switching from a high fat diet to normal chow in wild-type mice led to a reduced caloric intake on the first day with an increase to a steady state value that was calorically less than the intake on a HF diet. On normal chow, daily food intake of PVN Sim1 neuron ablated mice was 208% of that of the iDTR mice. On the first day after switching to HF, iDTR mice increased their gram food intake by 23% while Sim1creiDTR mice decreased their gram food intake by 17%, Sim1CreiDTR mice continued to decrease their intake such that by the second day of the HF diet, their intake was lower than their iDTR littermates. Over the following week on high fat diet, food intake of both iDTR and PVN Sim1 neuron ablated mice gradually decreased (Figure 5A). Surprisingly, average food intake in PVN Sim1 neuron ablated mice during the week on HF diet was 16.9% lower than iDTR mice. On day one after returning to normal chow, PVN Sim1 neuron ablated mice increased their caloric intake significantly (by more than 500% of their pre-HF intake). This increase raised their intake to a level similar to their intake prior to switching to HF diet. By contrast, iDTR mice exhibited a 30.8% decrease in gram food intake compared to the last day on HF diet and a 49.3% decrease compared to previous level on normal chow. After switching back to normal chow, both iDTR and PVN Sim1 neuron ablated mice gradually restored intake to that prior to diet change (Figure 5A). iDTR increased their caloric intake to 172% of their previous intake on the first day after switching to HF and decreased their intake by 50.5% when switched back to normal chow diet, with a slow return to their caloric intake prior to the switch occurring gradually during the following week (Figure 5B). PVN Sim1 neuron ablated mice increased their caloric intake to 121% of that on chow diet first day after switching to HF. After that first day, PVN Sim1 neuron ablated mice decreased their caloric intake on HF diet to a level falling below their intake on normal chow diet during the week (Figure 5B). During the week on a high fat diet, caloric intake of PVN Sim1 neuron ablated mice was lower than iDTR mice, a result that was the reverse of what we observed in these two groups of mice on normal chow diet. Average energy intake during the week on the HF diet was increased by 40% in iDTR mice, but decreased by 41.4% in PVN Sim1 neuron ablated mice. Body weight of both groups was examined daily. Consistent with previous studies, we found that iDTR mice showed an increase of body weight on HF diet, and then a decrease after being switched back to normal chow (Figure 5C). Conversely and surprisingly, PVN Sim1 neuron ablated mice exhibited a decrease in body weight on the HF diet, and then a regain of weight upon being returned to a chow diet (Figure 5C). These results indicate that PVN Sim1 neuron ablation was associated not only with hyperphagic obesity on normal chow, but, unexpectedly, with hypophagia and weight loss on a HF diet. 

### PVN Sim1 Neuron Ablation leads to decreased preference for HF diet and decreased overall intake in the context of HF food

To better understand the effects of the HF diet in PVN Sim1 neuron ablated mice, we performed food choice experiments comparing their food preference to that of iDTR mice in the context of availability of both high fat and normal chow diet (Figure 5D). As might be expected given their decreased intake on a HF diet, PVN Sim1 neuron ablated mice exhibited a strong preference for normal chow over HF food in contrast to iDTR mice. The availability of a HF diet, unexpectedly, led to a decrease in overall caloric intake in PVN Sim1 neuron ablated mice such that their overall caloric intake was (a) significantly decreased relative to their intake on a chow diet and (b) there was no significant difference in intake between them and their iDTR littermates (Figure 5E). 

### Gene Expression Analysis following Ablation of PVN Sim1 Neurons

Previous studies have found that the PVN and amygdala are involved in the preference for and sensitivity to carbohydrates or a high fat diet [14,25-27]. In these nuclei, others have observed that melanocortin (MC) and galanin (GAL) signaling play an important role in the regulation of food preference [4,10-12,14,16,28]. To investigate the mechanisms for the resistance of mice with PVN Sim1 neuron ablation to HF diet, we examined mRNA expression of obesity-related genes in these two brain regions. We found that SIM1 and MC4R mRNA abundance were decreased by 73.3% (p< 0.05) and 13.7% (p< 0.05) respectively in the PVN relative to iDTR mice (Figure 2A and 2B). We were surprised at the small magnitude of MC4R mRNA decrease in the PVN, so to investigate this phenomena further we examined the relative abundance of OXT mRNA in the PVN (Figure 2C). Oxytocin mRNA showed a significant decrease of 86.7% in the PVN in Sim1 neuron ablated mice relative to iDTR mice (p<0.05). 

Concurrent with these decreases in the PVN, we observed that SIM1 and MC4R mRNA abundance were increased to 333% of baseline (p< 0.05) and by 23% (p< 0.01) respectively in the amygdala when comparing PVN Sim1 neuron ablated mice to iDTR controls (Figure 2 E, F). There were no significant differences between GAL mRNA expression in either the PVN and amygdala between iDTR and Sim1creiDTR mice (Figure 2 G, H). 

## Discussion

In the present study, we examined the role of PVN Sim1 neurons in the regulation of food intake and energy expenditure. Our work confirms a critical role for SIM1 positive neurons in the PVN in the control of intake. Specific ablation of Sim1 neurons in the PVN results in profound obesity, increased food intake and decreased energy expenditure. Mice lacking PVN Sim1 neurons have a blunted response to a) AgRP –induced drive to eat and b) MC4R selective agonist- induced energy expenditure. These results are similar to those described in other genetic models of central obesity (e.g. SIM1^+/-^ and MC4R^-/-^ mice) [5,6,15,29,31]. In contrast to conventional deletion of SIM1 or MC4R, we found several novel, interesting and important results after PVN specific Sim1 neuronal ablation. Most notably, mice with PVN Sim1 neuron ablation exhibit decreased food and energy intake on a HF diet. This intake is decreased compared to either their intake on normal chow and to the intake of control iDTR mice on a HF diet. 

Previous work has noted that PVN ablation leads to hyperphagia on normal chow diet[3]. Here we find that Sim1 PVN neuron ablated mice appear to normalize their intake on HF diet. Thus, our work is the first to recognize this phenomenon and to describe one of the signaling pathways implicated (i.e. SIM1). As expected, in our SIM1 PVN neuron ablated mice SIM1 was dramatically decreased in the PVN. In concert with this change, SIM1 mRNA expression was dramatically increased in the amygdala. Given SIM1’s apparent role in negatively regulating appetite, this may indicate that Sim1 neurons in the amygdala play a critical role in regulating food intake on a high fat diet. In addition, the apparent reciprocal regulation of SIM1 in the amygdala implies that SIM1 plays a global role in the brain for overall nutrient sensing. It may be that SIM1 PVN neuron ablated mice are a good model for obesity associated with post-surgical hypothalamic dysfunction. In this context, advising post-surgical hypothalamic dysfunction patients to avoid fat may not be the appropriate approach; instead, our results suggest that encouraging them to include fat in their diet might lead to a decrease in overall caloric intake. 

Prior studies [10] strongly suggest that melanocortin signaling in the PVN controls food intake, while elsewhere melanocortin signaling controls energy expenditure. SIM1 haploinsufficiency leads to hyperphagia without altering energy expenditure [6,30]. Other work finds that global ablation of adult Sim1 neurons causes both hyperphagia and reduced energy expenditure, indicating that the homeostatic role of Sim1 neurons may be broader than the role of the SIM1 gene. 

MC4R is broadly expressed in many brain areas implicated in the regulation of energy balance [32]. Pharmacological and genetic studies have suggested MC4R signaling in the PVN regulates appetite and energy balance, however, the specific role of MC4R in PVN neurons, such as SIM1, remains unknown [33]. We found that mice with PVN Sim1 neuron ablation exhibit no response to the MC4R reverse agonist AgRP, despite a modest decrease in MC4R mRNA abundance in the PVN. Interestingly, MC4R expression in the amygdala was found to be modestly up-regulated in our model. Previous work has reported that melanocortin signaling in the amygdala controls appetite for dietary fat [14]. Our study is not-inconsistent with this data. 

**Figure 6:**
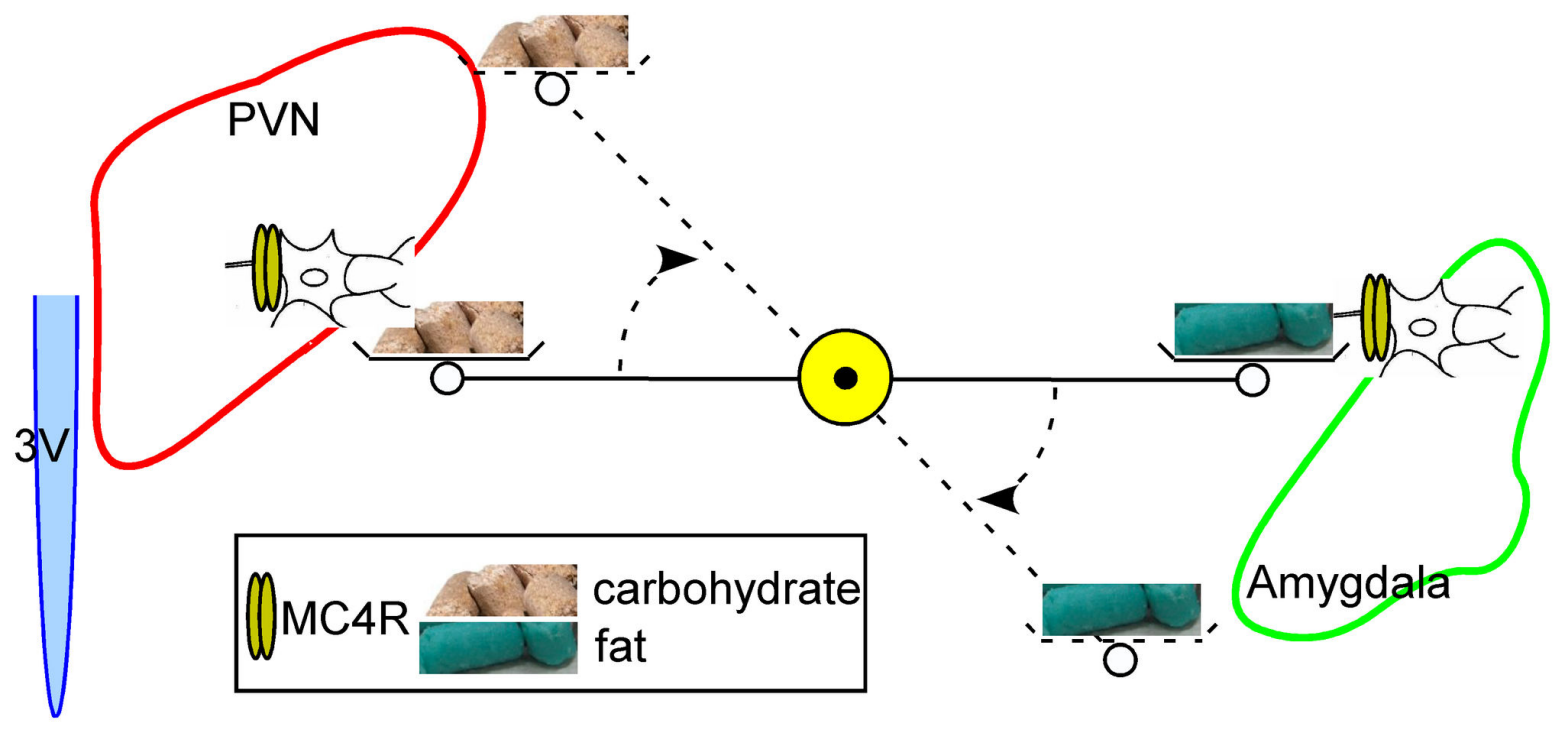
Schema depicting Sim1 as global regulator of intake. In this model loss of Sim1 neurons in the PVN leads to increased Sim1 mRNA transcription in the amygdala and increased sensitivity of the amygdala to the satiety inducing effects of fat.

This work suggests that upregulation of SIM1 expression at the amygdala in response to decreases in SIM1 expression in the PVN leads to decreased fat appetite. Given MC4Rs apparent role in fat appetite, this result would imply that MC4R signaling in anatomically separate nuclei (e.g. the amygdala and the PVN), associated with SIM1 expressing neurons, maintain overall energy intake of carbohydrate and fat. Based on these results we propose the following mechanistic model where deletion of Sim1 neurons in one region of the brain leads to an increase in another region in SIM1 transcription, and up regulation of signaling pathways downstream of Sim1 such as MC4R (Figure 6). 

Galanin (GAL) levels in the PVN are positively related to the preference of the animals for fat [26]. A direct relationship between GAL and the metabolism of fat is suggested by evidence that pharmacological blockade of fat oxidation reduces PVN GAL expression while suppressing fat intake [27]. We examined the interaction between galanin and SIM1. We found that PVN Sim1 neuron ablation had dramatic effects on appetite for fat, but did not significantly alter GAL expression in either PVN or amygdala. This result suggests that GAL functions via different pathways and neural sites from SIM1, or that PVN Sim1 neurons play a permissive role for GAL function on fat appetite. 
