# Correction: Paraventricular Nucleus Sim1 Neuron Ablation Mediated Obesity Is Resistant to High Fat Diet

**DOI:** 10.1371/annotation/9239a129-5677-43b0-8fe1-0c1e75e988df

**Published:** 2014-01-06

**Authors:** Dong Xi, Jeff Roizen, Meizan Lai, Nilay Gandhi, Bassil Kublaoui

The figure legends for Figures 2-5 have been posted out of order. The correct figure legends for each figure can be found below:

Figure 2: **Real-time quantitative PCR comparing Sim1 mRNA, MC4R mRNA, and Galanin mRNA in PVN and Amygdala of Sim1creiDTR and iDTR mice, and comparing OXTmRNA in PVN of Sim1creiDTR and iDTR mice.**

Relative mRNA abundance of SIM1 (A), MC4R (B), OXT (C), and GAL (F) expression in the PVN and SIM1 (D), MC4R (E), and GAL (G) amygdala. (n=4 to 9 for each group, * p< 0.05, ** p<0.01, ***p<0.001) .

Figure 3: **Body weight (A)(D), food intake (B)(E) and feeding efficiency of male and female Sim1creiDTR and iDTR mice.**

Body weight and food intake were measured weekly on a chow diet (n= 10 for male groups, n=9 for female groups, *p<0.05) (C) (F) feeding efficiency of male and female mice calculated as the ratio between weekly body weight change (g) and food intake (g) (n=8 for each group, * p<0.05).

Figure 4: **Food intake after 12 hour fast, food intake after AgRP injection and metabolic rate after MC4R agonist injection.**

(A) (B) 4 hour and 24 hour intake of normal chow after fasting in Sim1creiDTR and iDTR mice after overnight 12 hour food deprivation (n≥4 for each group). (C)(D) 4 hour and 24 hour intake of normal chow in Sim1creiDTR and iDTR mice after injection of AgRP at 20 pmol into the PVN (n≥4 for each group). (E)(F) Oxygen consumption and metabolic rate of Sim1creiDTR and iDTR mice at 0min, 15 min, 30min, 60 min, and 120 min after injection of MC4R selective agonist into the PVN (n=3 for each group). Energy expenditure was measured in CLAMS cages during light cycle. *p<0.05, **p<0.01, ***p<0.001.

Figure 5: **Response of Sim1creiDTR and iDTR mice to high fat diet.**

(A) Food intake and (B) energy intake of Sim1creiDTR and iDTR mice. Mice were fed with chow for a week, switched to HF for a week, then back to chow(n=6 for each group). (C) Average daily body weight change of Sim1creiDTR and iDTR mice when fed with a chow diet or HF diet (n=6 for each group). (D)(E)Daily food intake (E) and energy intake (F) of Sim1creiDTR and iDTR mice fed with mixed diet of chow and HF (n=7 to 9 for each group). * p<0.05.

